# Health policy counterpublics: Enacting collective resistances to US molecular HIV surveillance and cluster detection and response programs

**DOI:** 10.1177/03063127231211933

**Published:** 2023-12-06

**Authors:** Stephen Molldrem, Anthony K J Smith

**Affiliations:** 1 University of Texas Medical Branch, Galveston, TX, USA; 2 UNSW Sydney, Kensington, Australia

**Keywords:** HIV/AIDS, pathogen genomics, social theory, policy studies, social movements, publics

## Abstract

Health policies and the problems they constitute are deeply shaped by multiple publics. In this article we conceptualize *health policy counterpublics*: temporally bounded socio-political forms that aim to cultivate particular modes of conduct, generally to resist trajectories set by arms of the state. These counterpublics often emerge from existing social movements and involve varied forms of activism and advocacy. We examine a health policy counterpublic that has arisen in response to new forms of HIV public health surveillance by drawing on public documents and interview data from 2021 with 26 stakeholders who were critical of key policy developments. Since 2018, the national rollout of molecular HIV surveillance (MHS) and cluster detection and response (CDR) programs in the United States has produced sustained controversies among HIV stakeholders, including among organized networks of people living with HIV. This article focuses on how a health policy counterpublic formed around MHS/CDR and how constituents problematized the policy agenda set in motion by federal health agencies, including in relation to data ethics, the meaningful involvement of affected communities, informed consent, the digitization of health systems, and HIV criminalization. Although familiar problems in HIV policymaking, concerns about these issues have been reconfigured in response to the new sociotechnical milieu proffered by MHS/CDR, generating new critical positions aiming to remake public health. Critical attention to the scenes within which health policy controversies play out ought to consider how (counter)publics are made, how problems are constituted, and the broader social movement dynamics and activist resources drawn upon to contest and reimagine policymaking in public life.

## Introduction

The process of publicly challenging a policy often brings new political constituencies into being. In fact, it has become common sense in Science and Technology Studies (STS) to say that the contestation of a problem is constitutive of the problem itself, or that issues are only enacted as ‘political’ once they are successfully characterized and recognized as such (Callon et al., 2011; Law, 2009; Mol, 1999; Murphy, 2006). However, creating new territories for political struggle in policy is often difficult, particularly when arguing, from a subordinate or marginal position, against trajectories set in motion by arms of the state (Brown et al., 2012; Epstein, 1996; Hess, 2016; Rogers, 2022). The formation of publics centered on the *problematization* of policies in the public sphere also requires resources and sustained work by activists, state actors, experts, and other actors (on ‘problematization’, see Bacchi, 2012, 2016).

Increasingly, health policymaking processes are deeply shaped by social movements led by patients and people living with and affected by chronic conditions, rare disorders, and other diseases (Brown et al., 2019; Epstein, 2008; Keller & Packel, 2014; Murphy, 2006, 2012; Parthasarathy, 2007). As health policymaking has expanded as a site of struggle, contestations over specific policies have generated numerous new forms of collective political and technoscientific life (Epstein, 2008; Hess, 2011).

Since the 1980s, HIV/AIDS policy has been indelibly shaped by political dynamics between government initiatives and social movements led by people living with by HIV (Epstein, 1996, 2007; Fairchild et al., 2007). In this article we consider some sustained HIV-related advocacy in the United States since 2018. This advocacy has centered on challenging and reshaping federal policymaking processes related to a novel class of HIV public health interventions called ‘molecular HIV surveillance’ (MHS) and ‘cluster detection and response’ (CDR). MHS/CDR programs involve public health agencies re-using HIV genetic sequence data collected from people living with HIV during routine medical visits to guide epidemiological analyses and prevention outreach (Centers for Disease Control and Prevention [CDC], 2018; Molldrem & Smith, 2020; Oster et al., 2021). In the clinical context, HIV genetic sequence data help providers prescribe appropriate medications by identifying mutations in a person’s HIV that may confer antiretroviral drug resistance (CDC, 2018; Oster et al., 2021). When reported to HIV surveillance systems operated by departments of public health and analyzed using phylogenetic analysis to measure genetic distances between individuals’ HIV strains, HIV genetic sequences can reveal people living with HIV in ‘clusters’ of recent transmission; this is MHS (Cohen et al., 2014; Oster et al., 2021). CDR involves public health investigation and outreach activities using MHS data and other sources of information in which people in HIV transmission clusters and their partners are targeted for prevention, contact tracing/partner services, (re-)linkage to care, and other interventions (Molldrem & Smith, 2020; Molldrem et al., 2023b; Oster et al., 2021). Cluster response events can also involve public health departments communicating with media, posting about clusters online, and other forms of awareness-raising (CDC, 2018).

Critiques and other analyses of MHS/CDR programs by practitioners, bioethicists, legal scholars, civil society advocates, and people living with HIV have centered on the lack of informed consent requirements for uses of data in public health programs, as well as concerns about how MHS/CDR relates to the criminalization of HIV transmission and nondisclosure to partners in many US jurisdictions (Bernard et al., 2020; Center for HIV Law and Policy [CHLP], 2019; McClelland et al., 2020; Molldrem & Smith, 2020; Mutenherwa et al., 2019b; Sandset, 2020; Schairer et al., 2019). The successful framing of MHS/CDR as a problem in health policy is largely attributable to sustained advocacy about these programs since 2018—spearheaded mainly by organized networks of people living with HIV—that has aimed at stopping or reforming their rollout. Many competing positions have been put forward in debates over the future of these programs, ranging from the view that MHS/CDR activities pose no special ethical dilemmas and that the technologies that enable these programs are simply ‘old wine in new bottles’ (Dawson & Latham, 2020), to demands that HIV surveillance and prevention ought to be ‘abolished’ as currently practiced (Bernard et al., 2020). In response to these developments, the Presidential Advisory Council on HIV/AIDS (PACHA), the US’s highest federal HIV/AIDS advisory body, has issued a pointed call for major reforms to MHS/CDR (PACHA, 2022; see also Molldrem et al., 2023a).

Drawing on our prior research about the US MHS/CDR controversy, public documents, and 26 interviews with a diverse range of US-based HIV stakeholders who identified as critical or concerned about the implementation of MHS and CDR programs, we argue that this constituency’s sustained resistance and advocacy activities, aimed at stopping or reforming MHS/CDR, have led to the formation of what we call a ‘health policy counterpublic’. We define health policy counterpublics as impermanent collective entities that form around specific policy controversies, resisting trajectories set in motion by arms of the state with the explicit aim of changing the direction of a particular area of health policy. Health policy counterpublics often emerge from pre-existing social movements but are also open-ended and involve other actors who take positions critical of the state within a given controversy, thus becoming actors within the health policy counterpublic in question. These counterpublics take shape by articulating specific policy goals in time-bounded controversies and enact strategies aimed at changing health policies by organizing to make appeals for reform or transformation. They thus desire, seek, and gain recognition from the state—and when the policy controversy at issue resolves, the health policy counterpublic(s) specific to it also necessarily dissolves or transmutes into something else (see Murphy, 2012). A counterpublic might generate concern around matters related to health and even health policy, but if it does not form around seeking to change policy and successfully secures recognition from the state toward that end, it is a different sort of public than what we are calling a health policy counterpublic. Thus, not all health-oriented counterpublics with interests in changing health policy materialize as health policy counterpublics.

The US MHS/CDR controversy is an exemplary case where a health policy counterpublic has emerged. We discuss how it formed through activist and academic networks starting in 2018, and how it has problematized ethical norms in public health and the policy frameworks that support public health practice, thereby also reshaping the trajectory of US HIV public health policy.

In the following section we situate our conceptualization of health policy counterpublics within key literatures in STS, social movement studies, and social theory. In doing so, we aim to differentiate the concept from other related analytics and to argue for the specificity of health policy counterpublics as a particular kind of socio-political form.

## Health policy counterpublics

### Publics

Our approach draws on theories of publics that emphasize their ephemerality, open-endedness, and ability to act toward certain collectively-desired ends (Bourdieu, 1993; Fraser, 1990; Lippmann, 1993[1925]; Warner, 2002). For theorists of politics and the public sphere who inspire this framing, such as Chantal Mouffe, Walter Lippmann, Carl Schmitt, and Antonio Gramsci, ‘politics’ and ‘the political’ are conceptualized as conflicts between aligned and opposing interests across state, industry, and civil society that unfold through what Gramsci calls ‘wars of position’ or ‘wars of movement’ where differing factions struggle for—and eventually achieve—dominance in their areas of interest and concern (Crooks & Currie, 2021; Gramsci, 1992[1929-1935]; Laclau & Mouffe, 2001[1985]; Schmitt, 2007[1932]). These understandings of publics and politics are opposed to the idea—central in many dominant approaches to the study of liberal-democratic governance—that anything like ‘the public’ or ‘public opinion’ exists prior to interest groups mobilizing their constituencies around particular issues (see also, Bourdieu, 1993; Law, 2009; Lippmann, 2012[1922]). Our approach is intentionally positioned against the possibility of realizing an idealized ‘Public’ or ‘Great Community’, in the mode of John Dewey—a fantasy of a harmonious polity that can be worked into existence through democratic exercises of rational-critical debate that aim to generate enduring common feeling or consensus-based positions across society (Dewey, 1991[1927]; Habermas, 1985). Rather, our analysis of conflicts over health policy and the actions of health policy counterpublics centers the generative nature of political conflict within and beyond the pluralist public sphere, emphasizing the importance of confrontations between interest groups and the formation of strategic alliances that aim at taking power as much as they seek to engage in rational-critical deliberation (Laclau & Mouffe, 2001[1985]). In this approach, invocations of ‘public opinion’, ‘public benefit’, or ‘the public interest’ by interest groups or arms of the state are understood as examples of what Pierre Bourdieu called ‘mobilized opinion’ or ‘formulated opinion’ directed strategically by ‘pressure groups mobilized around a system of explicitly formulated *interests*’ (Bourdieu, 1993, p. 157, emphasis in original). The public sphere and the terrain of politics are thus battlegrounds for affective forms of politics as much as they are forums for rational-critical argument (Gould, 2009).

### Counterpublics and health policy

The concept of a health policy counterpublic is informed by theories of *counterpublics*—a form of subaltern collectivity that, per Michael Warner, necessarily ‘maintains at some level, conscious or not, an awareness of its subordinate status’ to dominant forms of opinion that structure society and most political conflict within it (Warner, 2002, p. 56; see also Fraser, 1990). As Warner writes, counterpublics are social forms that only tend to become oriented toward outwardly directed action when they self-organize around a shared desire to affect the state by engaging in conflicts over policy from this self-aware subaltern positionality. He notes that

it might be only through its imaginary coupling with the state that a public acts. This is one of the things that happens when alternative publics are said to be social movements: they acquire agency in relation to the state. They enter the temporality of politics and adapt themselves to the performativities of rational-critical discourse. (p. 124)

A health policy counterpublic is this sort of counterpublic: an open-ended socio-political form that facilitates the circulation of discourse about a contested topic in health policy amongst actors who are trying to affect policy from a self-aware subordinate position to dominant paradigms set mainly by arms of the state.

Importantly, health policy counterpublics’ general tendency toward resistance to policy trajectories set by state agencies does not imply that they are *entirely* external to the state. Indeed, as explored later, the MHS/CDR health policy counterpublic has been deeply interwoven with the state, including individuals employed or contracted by state and local public health agencies, and individuals in academia and community-based organizations who receive funding from federal, state, and local government agencies. Health policy counterpublics can thus be both *other than the state* while also being *interwoven with the state*. However, they are necessarily what Nancy Fraser calls ‘weak publics’—a form of (counter)public that she counterposes to ‘strong publics’ vested with authority to make decisions that can acquire force of law, such as legislatures or regulatory panels (Fraser, 1990, pp. 74–77). Health policy counterpublics are weak publics because they do not possess decision-making authority or even consistent state recognition; they instead mainly work to affect decision-making through activism, advocacy, and participation in policymaking, including through rational-critical discourse. Health policy counterpublics thus seek and secure some level of state recognition as legitimate policy actors, and some members of health policy counterpublics may even work mainly within the state apparatus. These counterpublics strategically mobilize limited resources and a variety of aligned interests on the terrain of political struggle within shifting wars of position (Gramsci, 1992[1929-1935]; Laclau & Mouffe, 2001[1985]).

In contrast to community-level practices of ‘counterpublic health’ (see Race, 2009, pp. 157–163), which are chiefly modes of counter-conduct in programmatic public health activity and on-the-ground public health work (e.g. Duff & Moore, 2015), a health policy counterpublic is mainly organized around advocacy, consciousness-raising, and trying to affect the direction of an area of health policy against courses of development already set by public agencies. Health policy counterpublics are what Warner characterizes as ‘closer … to politics’ (Warner, 2002, pp. 96–97) than counterpublics-in-general, and necessarily take an oppositional stance in relation to the positions of relevant agencies charged with policy-setting and implementation (see Fraser, 1990). It thus follows that, if a particular health policy counterpublic ceases to take an adversarial stance toward the state or if its members’ preferred policies are adopted *as policy*, a health policy counterpublic either dissolves or transmutes into some other kind of social form. Murphy (2012) has described how this occurred in the case of subaltern feminist self-help counterpublics of the 1960s-70s, which began as countercultural formations, shifted into making appeals aimed at the state (perhaps becoming health policy counterpublics), and later became incorporated into the global biopolitical apparatus of mainstream state-funded ‘women’s health’ programs.

### Problematizing policies and illuminating controversies in science and health policymaking

Within STS, theories of publics, counterpublics, and health-oriented social movements assist in understanding how policies materialize and how they enact particular realities (Irwin, 2006; Murphy, 2006; Welsh & Wynne, 2013). In idioms favored by Bacchi and Latour, health policy counterpublics take issues in health policy and ‘problematize’ them, turning ‘matters of fact’ in the governance of health into ‘matters of concern’ that can be debated and worked with on new terms (Bacchi, 2012, 2016; Latour, 2004). In the formation of a health policy counterpublic, through exchanges of discourse among members and the contestation of facts, practices, and norms in the public sphere a health policy problem comes to matter in a way that it did not before, thus reshaping the war of position around the matter of concern.

Further, health policy counterpublics have affective qualities; they draw on, mobilize, and capacitate different sentiments and moods that motivate action (Gould, 2009; Murphy, 2012). The people in a health policy counterpublic are engaged in political struggle over what are often highly technical matters, but also core principles that invoke strong feelings, because they care about the matter of concern and want it to be different—both ‘in reality’ and in its treatment in policy (Ritter, 2020). Health policy counterpublics form or ‘materialize’ in order to affect the ontological status of an underlying issue that policy affects by (re-)articulating the terms upon which that policy can be contested on the terrain of politics (Mol, 1999; Murphy, 2006; see also Pfaffenberger, 1992). They enable subaltern actors to coalesce on a matter of imminent concern to better problematize *collectively* in public.

### Social movements and health policy counterpublics

Health policy counterpublics differ substantially from other more enduring subaltern social forms or organizations interested in changing the trajectory of health policy. This is because they materialize around specific controversies and aim to make changes to particular policies or policy trajectories as those policies are being formulated or implemented by the state, often in concert with actors in civil society. While existing social movements may help provide part of the foundation for a health policy counterpublic to emerge, health policy counterpublics are not analogous to, for example, the ‘emergent concerned groups’ elucidated by Callon and Rabeharisoa or to what Brown and colleagues call ‘embodied health movements’ (Brown et al., 2019; Callon & Rabeharisoa, 2008). These entities and other iterations of what Epstein calls ‘patient groups and health movements’ are long in their duration, often aim to become entrenched interest groups, and are generally focused on an entire field of action in biomedicine and health rather than a limited set of health policies being enacted in a particular period (Epstein, 2008). For example, the people living with HIV/AIDS movement officially started in 1983 with the publication of ‘The Denver Principles’ and has since established itself as a key player across a range of sectors in US domestic and global health policy (People with AIDS Advisory Committee, 1983; Spieldenner et al., 2022). That movement certainly played a central role in the emergence of the US-based MHS/CDR health policy counterpublic of 2018–present.

The MHS/CDR health policy counterpublic is a specific entity formed in response to specific policy developments. The fact that the MHS/CDR health policy counterpublic emerged partly from existing social movements that have long shaped US HIV policymaking and science-making thus places it in the borderlands between what Welsh and Wynne distinguish between ‘uninvited’ versus ‘invited’ publics (Welsh & Wynne, 2013). However, it remains a *counter*public (rather than simply a *public*) because of its self-aware subordinate position and opposition to policy trajectories set in motion by the state.

While health policy counterpublics can include scientists and other health experts, they are not fully coextensive with the sort of ‘scientific counterpublics’ discussed by Hess (2011, 2016). Hess uses this concept to describe the role of marginal voices within communities of scientific practice—for example, researchers in areas such as cancer who work outside of dominant paradigms supported by national funding bodies and philanthropies. Health policy counterpublics do not necessarily *only* include voices from the margins of science—the MHS/CDR health policy counterpublic, for example, has engaged many mainstream scientific and policy voices (PACHA, 2022; Positive Women’s Network-USA, 2022; U.S. People Living with HIV Caucus, 2020). Further, like scientific counterpublics, health policy counterpublics tend to engage in forms of ‘scientism’ in their efforts to alter the trajectory of health policy (Welsh & Wynne, 2013). They do so partly by making appeals to scientific evidence, ‘the public interest’, historical practices, ethical norms, civil and human rights, democratic values, robust community involvement, greater stakeholder engagement, and other areas that operate on the plane of rational-critical discourse in science and technology policymaking. These forms of ‘public talk’ are crucial (and perhaps even necessary) avenues for health policy counterpublics to pursue in order to achieve success in the current milieu of technoscientific governance (Irwin, 2006). In this kind of war of position, an ‘outsider’ strategy of confrontational activism alone can only be partly successful, as shown by the history of insider/outside activism in the history of AIDS (Epstein, 1996, 2007, 2008; Gould, 2009). If reshaping policy is the goal, being able to speak the languages of policy and to operate legibly and effectively within the health policy scene and corridors of power is, to some degree, required. Health policy counterpublics traverse these borderlands of confrontational strategies and demands for inclusion in official policymaking processes. Examples of other movements that have used insider-outsider strategies to affect particular policies include feminist counterpublics that argued for the existence and official recognition of ‘sick building syndrome’ (Murphy, 2006), chronic fatigue activists who have sought recognition by state and biomedical actors (Rogers, 2022), and the campaign to redefine the CDC definition of AIDS to be more inclusive of women that succeeded in 1993 (Shotwell, 2014).

To summarize, health policy counterpublics self-organize with varying degrees of intentionality in attempts to shape health policymaking in directions aligned with their self-understood interests. They operate from a self-aware subaltern position, but also seek recognition from the state and aim explicitly to affect particular health policy trajectories that have been set in motion by arms of the state and dominant actors in civil society. The temporal nature of the MHS/CDR controversy, unfolding only since 2018, makes an ideal case study for the elaboration of the concept of health policy counterpublics.

## The study: Understanding criticisms and concerns about MHS among HIV stakeholders in the US

This study aimed to explore the perspectives of critics of MHS/CDR in the US HIV response. To be eligible, participants needed to self-identify during recruitment as a US-based HIV stakeholder with criticisms or concerns about MHS/CDR. We sought participants with varied expertise in the HIV sector, and who represented perspectives across public health institutions, civil society, advocacy groups, and organized networks of people living with HIV. SM, the first author, invited participants via email using an initial small sample drawn from his knowledge of the HIV sector, followed by snowball sampling using confidential referrals. All participants provided verbally recorded consent before interviews. This study was considered exempt human subjects research by the University of California (UC), Irvine Institutional Review Board. Study funding was provided by the UC President’s Postdoctoral Fellowship Program, through SM.

The sample included 26 participants who were demographically diverse, highly educated, and had had long-term involvement and expertise in the HIV/AIDS sector, including as activists, public health professionals, or researchers, with many inhabiting multiple roles over time (for further discussion of participant demographics, see Molldrem, Smith, & Subrahmanyam, 2023). All participants held expertise about the US HIV response, including traditional forms of scientific or public health expertise, policy expertise, and types of ‘lay expertise’ that have shaped the HIV/AIDS response (Epstein, 1996). Many participants had been involved in previous US HIV/AIDS policy developments, including criminal law reform, the transition to names-based case reporting in the 1990s and 2000s (Fairchild et al., 2007), and shifts in HIV prevention stemming from knowledge about HIV treatment-as-prevention. In reporting quotes, we have categorized participants into a single dominant ‘role’ (e.g. [P01—Researcher]).

SM developed an interview guide and conducted qualitative semi-structured interviews with participants between January and May 2021 over a video conferencing platform. Participants were asked questions about their work and expertise in the HIV sector, their perspectives about the rollout of MHS and CDR, their specific criticisms and concerns, key topics such as consent, criminalization, and community engagement, followed by demographic details. On average, interviews lasted 1 hour and ranged from 33 to 79 minutes. SM made notes during interviews and wrote reflective memos after each. Interviews were audio recorded, professionally transcribed, checked for accuracy, and deidentified to ensure confidentiality.

We analyzed interviews using reflexive thematic analysis, an iterative approach that draws on researchers’ subjectivity, research questions, and understandings of the literature to continuously reflect on assumptions through the process of analyzing interview material (Braun & Clarke, 2019). In this approach, themes are patterns of meaning developed through analysis, which includes a process of coding, reviewing literature, refining the conceptual focus, and writing. In approaching the analysis for this paper, we aimed to explore how scientific controversies are constituted, expressed in policymaking, and also focused on understanding the variety of critiques about MHS/CDR within a critical constituency.

In executing data analysis, SM familiarized himself with the data, including listening to audio recordings, reading transcripts, and writing reflective memos about the study data and the overall analytical process. He conducted several rounds of iterative coding in Atlas.Ti software, including codes that repeated across multiple interviews and which captured broad concepts. AS also read each transcript and made notes, and both authors met on a weekly basis for several months while SM coded the data to discuss interpretation and initial theme generation. Our initial analysis focused on identifying the range of positions that participants took about key aspects of MHS/CDR. Given that participants’ opposition to MHS/CDR primarily centered on critiques of its rollout by federal, state, and local government agencies, and that participants were involved in a range of activities seeking to reform or challenge MHS/CDR, we came to understand their positions, concerns, and organizational form as a counterpublic.

We considered several interrelated questions: How did contestations over MHS/CDR take shape? Who was involved, what kind of actions occurred, and on what bases were actions taken? Further, what aspects of MHS/CDR were problematized and what alternative policy futures were imagined? Our analysis of this case led us to the general health policy counterpublic concept.

## The MHS/CDR health policy counterpublic

### Enacting a health policy counterpublic

A formative event for the MHS/CDR health policy counterpublic appeared to be a key meeting that took place during a national HIV community-led conference in 2018. Multiple participants cited this session and described what they expected to be a small side panel become a major part of the conference and a launchpad for sustained activism about MHS/CDR. In the words of P14, an advocate affiliated with organized networks of people living with HIV (hereafter, ‘PLHIV network’),

50 or 60 people showed up … lawyers, people living with HIV, people of trans experience, Black and brown people, people just really concerned that this had been rolled out. … We had to become experts very quickly in it—you know, it reminded me of when you first get HIV, and you have to suddenly become an expert in your medical care even though you have no training for it.

According to another participant, ‘The room ended up being packed, it was standing room only. From there is where the activism and advocacy and organizing on MHS, primarily led by people living with HIV, really took off’ (P07—Advocate, PLHIV network). The momentum created by this meeting was palpable for other participants. In the words of another who mainly spoke about advocacy around issues related to HIV, the Latinx community, and undocumented persons:

We gathered and learned about the HIV Molecular Surveillance rollout, and since that day we have been meeting, a group of us, on a regular basis. We are the ones that have made the decision to call for a moratorium, because we think that CDC could have engaged much better with the communities that are most at risk. … I’ve directly asked the CDC: ‘What is your plan to prevent poor immigrants that are maybe going to be deported eventually, would you share [MHS] information with ICE [Immigration and Customs Enforcement], for example?’ They’ve not been able to answer that question, and the fact that they could do this without answering that basic question, in my opinion, is just irreverent. So, it is certain organizations that have voiced a moratorium. [But] it is also the work that we’ve done behind the scenes. It’s a group of us that are actually concerned about our communities potentially harboring more distrust. (P15—Advocate, PLHIV network)

This key serendipitous event spurred a shift in advocacy priorities and was made possible by existing resources and infrastructures to support HIV community organizing. P15 also highlights how advocacy related to MHS/CDR has intersected with other social movements, including in relation to organizing against immigration authorities.

Opening up spaces for internal and external deliberation is a key function of politically oriented counterpublics (Brown et al., 2019; Fraser, 1990; Murphy, 2006, 2012; Rogers, 2022). Health policy counterpublics are not just an organizational form in the mode of singular non-profit agencies, political action committees, or advocacy coalitions. Rather, they are enacted through a collection of widely varying styles of engagement with the health policymaking process by a variety of actors and groups, partly through the intentional creation of discursive zones and opportunities for exchanging discourse that can be drawn upon and mobilized to build up a set of counterarguments (Pfaffenberger, 1992). Through sustained work by actors in the counterpublic within the broader health policy scene, health policy counterpublics acquire a kind of agency, become legible as policy actors, and are recognized by at least some relevant state agencies with authority over their area of concern.

Participants spoke about their goals of rapidly altering the trajectory of the MHS/CDR rollout and policymaking processes. These aims were often communicated with explicit reference to strategic conversations with other stakeholders, ongoing meetings, as well as moments of targeted advocacy in both public and private confrontations with the CDC and other federal agencies. The MHS/CDR health policy counterpublic emerged in relation to multiple social movements—including the HIV/AIDS response, people living with HIV and AIDS activism, the LGBTQ movement, the immigrant rights movement, and anti-racist social movements, all of which provided supportive infrastructures for MHS/CDR advocacy (on inter-movement dynamics in HIV/AIDS activism, see also, Epstein, 1996; Gould, 2009). Connections to one or more existing social movements help health policy counterpublics acquire legitimacy in the eyes of the state, moving them closer to deliberative processes central to contemporary policymaking and participation in the machinations of the liberal-democratic public sphere (Fraser, 1990; Laclau & Mouffe, 2001[1985]; Warner, 2002, pp. 96–97). The meeting described above operated in this way—as an important space and key moment for people who were differently positioned within the US HIV policymaking ecosystem to air out and deliberate over differences of viewpoint about MHS/CDR:

We had 30 or 40 people there, and they were sort of like all the real leaders in the community. That was absolutely the emergency meeting. It was ridiculous because everyone had heard a little something about this. The people who were the kind of community members who either work for big agencies or work for big parts of the government were like ‘No, no, it’ll be fine. It’s okay. The CDC is rolling this out.’ It actually was a really interesting and very emotional meeting. Half the people were like ‘Oh yeah, this is really interesting.’ The rest of us were like ‘Oh no, this is really scary. This is really, really scary.’ There were some people who hadn’t heard about it, and they were just like—the common reaction is ‘No, this can’t possibly be happening. …’ It was like, we started saying ‘This is in all health departments now, and this has been going on since 2013?’ In that room we had people who had been on [a major federal HIV/AIDS advisory body] at that time …. Like these are people who are involved in providing feedback to federal partners who have worked on federal grants, who have been really in the know, who were Senior Vice Presidents at large groups that didn’t know about it. So, it was just like, ‘First off, how did we not know about this? Why did somebody not bring it up?’

Again, it just goes back to this thing—‘Oh, we’re just going to look at this data. Don’t worry. We don’t really need to coordinate with people living with HIV because it’s just data, right?’ To the CDC it looks like it is just numbers, and data points, and neutral, and bland, and beige. To us, it’s people and lives. So, they don’t see a need to even talk to people living with HIV. … Why did nobody think we needed to know about it? What do we do now? So, that was the disbelief around it—it was like, ‘It can’t be legal. It can’t be possible. It can’t be really this ingrained. It can’t have gone on that long.’ (P16—Advocate, PLHIV network)

The mood of this meeting and the discursive space it opened laid the foundation for subsequent advocacy and the ways in which the problematization of MHS/CDR policies took shape within this counterpublic.

For a counterpublic to materialize as a health policy counterpublic, individuals and institutions which are members of the counterpublic in question must be recognized by state entities as legitimate participants in health policymaking processes. This also means that not every counterpublic organized around health or even around key topics in health policy is a health policy counterpublic. Said otherwise, just as there are barriers to membership in counterpublics that require recognizing oneself as a member of a subordinate social group (Fraser, 1990; Murphy, 2012; Warner, 2002), there are structural barriers for counterpublics to materialize *as health policy counterpublics*. The main such hurdle is some level of state recognition as a legitimate actor in health policymaking; without this, a social form might be a counterpublic or some other kind of dissident public, but it is not a health policy counterpublic.

The MHS/CDR health policy counterpublic has consistently gained state recognition, perhaps most notably in the 2022 resolution calling for reforms to MHS/CDR programs that was passed by PACHA, the highest federal advisory body on matters related to HIV policy—a resolution which was followed by a response from the White House Office of National AIDS Policy (ONAP) in March 2023 (Molldrem et al., 2023a; PACHA, 2022; Phillips, 2023). However, several years before this, in 2019, protests about MHS/CDR directed at then-CDC director Robert Redfield played a key role in advancing the counterpublic’s policy agenda (Molldrem & Smith, 2020). In the words of P14, an advocate with PLHIV networks,

We organized a protest because we had Redfield at the event. We used it as a target of opportunity to start protesting him. We also organized a protest of him at [another event] that same year. What it did was it caused him to realize there was a challenge, and on the CDC side, the surveillance people finally got a meeting with him because they had been saying they needed a meeting with him about this—the fact that a community conversation that needed to happen. At the time, he was very concerned.

This health policy counterpublic mobilized protracted forms of public protest—including protests in the aesthetic and confrontational traditions of radical AIDS activism—along with scientific publication, blasting out press releases, circulating petitions, issuing major reports, filing public comments to state agencies during designated periods where public input was sought, stakeholder engagement sessions, and conversations in arenas of health policymaking accessible only to those with some degree of insider access (Epstein, 1996, 2007, 2008; Keller & Packel, 2014). Along with capacity-building around the issue within the community of critical stakeholders, this diversity of tactics has been crucial to the measure of success that the MHS/CDR health policy counterpublic has had in affecting the trajectory of policy (Molldrem et al., 2023a).

In the following sections we turn to both how the MHS/CDR health policy counterpublic took form and the major issues that emerged from debates about key issues and organizing strategies within the counterpublic. These include issues raised above by P16, particularly concerns about the lack of meaningful engagement with people living with HIV in the rollout of MHS/CDR, and also how data are used, shared, and acted upon in public health systems in ways that potentially fail to consider how data relates to personhood, systems of domination, and forms of marginalization. What emerges is a clearer picture of how policy issues were problematized by the MHS/CDR health policy counterpublic, including concerns about MHS/CDR and broader critiques about how the US HIV epidemic is being handled in relation to social and material inequities.

### Fractured trust and partnerships: Implementing policy without meaningful consultation

As noted by the authors of a report stemming from a gathering of HIV stakeholders in 2017, MHS/CDR programs were scaled up very rapidly (Evans & Benbow, 2018, p. 12). The requirement for states to implement MHS and CDR programs was announced in an integrated HIV surveillance and prevention funding announcement released by CDC in 2017, which mandated that states begin undertaking these activities as part of the next round of funding (Centers for Disease Control and Prevention, and National Center for HIV/AIDS, Viral Hepatitis, STD, and TB Prevention [CDC and NCHHSTP], 2017). The structure and timeline of the announcement allowed very little time for community and stakeholder engagement about the implications of MHS/CDR (see Evans & Benbow, 2018; Spieldenner et al., 2022). The fallout from this decision by CDC involved a range of counter-reactions at several levels. Participants in our study who were employed by state and local health departments explained that they did not have a chance, before the rollout began, to voice their concerns or discuss MHS/CDR with either CDC personnel or their jurisdictional community advisory boards. Others in organized networks of people living with HIV described feeling blindsided and angered that they were not informed more systematically. Some policy professionals and researchers whom we interviewed took it upon themselves to explain MHS/CDR and to spur community engagement initiatives around the programs. One policy professional aimed to bring ‘together a diversity of stakeholders to think about this issue’ and that during those discussions, ‘we learned that community engagement wasn’t happening, because the first time communities [were] hearing about this [was] through us’ (P18—Advocate). Relatively fast responses by policy actors following what was perceived to be inadequate stakeholder engagement by CDC highlights the critical temporal dimensions that shaped the MHS/CDR health policy counterpublic’s formation around this specific controversy.

Implementing MHS/CDR nationally without meaningful community and stakeholder consultation was a key point of frustration and disappointment, and it structured the affective momentum of this health policy counterpublic. One public health professional who worked for a state-level public health agency explained that the loss of trust generated from a lack of engagement prior to rollout had resulted in a ‘schism’ between public health and HIV advocacy groups:

The biggest concern I have is the schism that it has developed between advocates and public health. We have a common goal here: to stop transmission of HIV, and we should be partners in that, but molecular surveillance has really changed that. I have never really seen, in my 30 years of working with this, this level of discord. Well, since [controversies over] name-based reporting [in the 1990s and 00s]. Maybe I should say since then. (P17—Public Health Professional)

Many participants shared feelings of anger and shock upon discovering that phylogenetic analyses of HIV genetic sequence data reported to HIV surveillance systems were being conducted without notifying people living with HIV that clinical data were being re-used in this way. P21 (Advocate, PLHIV Network) explained: ‘The community just did not know this was happening. … It was a shock, and it really hit a peak in terms of the controversy when the *Ending the HIV Epidemic* plan was rolled out [in 2019] and [MHS/CDR] was listed as one of the pillars’ (Department of Health and Human Services [HHS], 2019; Fauci et al., 2019).

Some participants reported an erosion of trust and transparency emerging from the MHS/CDR rollout. P14 (Advocate, PLHIV Network) explained:

Even now, the engagement is very nominal that the CDC has done. They’ve done one set of virtual townhalls. They’ve encouraged health departments to do some stuff, but basically the entire attitude is that ‘The train has left the station.’ You know, ‘No takebacks.’

This perceived lack of transparency in the MHS/CDR rollout led some participants to express their disillusionment over the commitment to community consultation in public health: ‘It has made me think about the dedication of public health professionals to communities. Is that really what the interest is?’ (P01—Research). For P24 (Public Health Professional), the rollout reinforced existing distrust that structures relationships between public health agencies, affected communities, and HIV advocacy groups, but was nonetheless characterized as an egregious example of non-consultation. They pointed out that community trust in public health was difficult to earn and maintain. ‘It is very easy to lose that trust, and once you do, it takes years to build it back.’

A central mobilizing aspect of this health policy counterpublic was the principle of meaningful involvement of people living with HIV, which the rollout of MHS/CDR was seen as undermining (see AIDS United & US People Living with HIV Caucus, 2018; Bernard et al., 2020). As P14 articulated: ‘The CDC has gone backwards in its engagement with people living with HIV just by implementing it. That’s a problem because without people living with HIV, you’re never going to end the epidemic.’ The emphasis on the lack of consultation by CDC and other agencies was repeated across interviews, and it became a unifying point of dissent about the MHS/CDR rollout that transmuted into a set of concrete demands.

### Not yet evidenced: Contesting the benefits and impacts of MHS/CDR

Proponents of MHS/CDR, including CDC staff involved in planning and implementation, have argued that the benefits are clear and established and that the programs will be useful in informing HIV prevention (CDC, 2018; Oster et al., 2021). In contrast, critical public health scholars and organizations representing networks of people living with HIV have criticized what they claim is an inadequate evidence-base for MHS/CDR (Bernard et al., 2020; HIV Justice Network & Positive Women’s Network, 2020; Molldrem & Smith, 2020; Positive Women’s Network, 2021; U.S. People Living with HIV Caucus, 2020). Across our interviews, participants held mixed views about the benefits of MHS/CDR and the quality of evidence supporting it. Stances ranged from calling for an immediate and indefinite moratorium on MHS/CDR, to arguing for further evidence to show the effectiveness and risks and benefits, to generally acknowledging the benefits of MHS/CDR but with specific concerns about how MHS may be mobilized (e.g. related to criminalization).

Participants differed greatly in whether they anticipated benefits from MHS/CDR. P04 (Public Health Professional) questioned the usefulness of MHS/CDR in practice:

Theoretically the CDC thinks that linking cases together helps you find a recent cluster that you could then dive into more deeply with proper services or other activities to find and diagnose people in that cluster and that would be helpful to identify people in that cluster who are not virally suppressed. It assumes that when you find this group of people who are genetically sequenced together that there is some level of recency [of infection], right? Because if you find people who are genetically clustered together, but all that infection happened 10 years ago, it’s kind of not relevant, right?

Others questioned whether MHS/CDR added unique benefits over established, evidence-based HIV prevention interventions, including traditional (i.e. non-molecular) epidemiological surveillance and prevention programs. ‘A lot of the clusters we find are just clusters that we could have identified ourselves, just by logically thinking and from what we already know, so I don’t see it as super beneficial in ending the epidemic’ (P03—Public Health Professional). Another participant asked, ‘Why do we know that there are thousands of people living with HIV living in [a suburban county], but there are still so few treatment options there, even while there’s molecular surveillance? I just really feel like I don’t see the impact’ (P13—Advocate, PLHIV Network). Building on an often repeated argument that disparities related to the HIV epidemic are well understood but poorly responded to (Bowleg et al., 2022; Philbin & Perez-Brumer, 2022), a few participants reported frustration with the claim that MHS/CDR was needed to act on these well-known issues: ‘I feel like there’s a high burden of proof to enact the most minimal intervention, like we need all this data just to justify helping Black people, honestly’ (P02—Advocate, PLHIV Network).

Although some participants were highly critical or suspicious of benefits, there were also participants who believed some benefits were clear. For example, P05 (Public Health Professional) explained: ‘It’s a very potent public health tool. There’s no getting around that. It is. The question is how you use it in a way that is not demeaning to people, dangerous to people, alienating to people.’ Similarly, according to P08 (Researcher):

I totally see the added value of phylogenetic research and molecular surveillance. It could move the field of prevention and care forward, I mean the studies have documented it. … Where we have failed is to inform the communities—people living with HIV—about the importance and significance of this research.

Notably, these participants highlighted key risks and concerns alongside benefits. Overall, most participants countered the notion that the benefits of MHS/CDR were clear and well known, and wanted to see further evidence of benefits and risks of MHS/CDR. However, a few participants, particularly advocates involved in organized PLHIV networks, wanted to see a moratorium on MHS/CDR until further evidence was generated (see also Positive Women’s Network-USA, 2021). In the words of one participant: ‘They shouldn’t go forward until a review and airing of the issues and resolution of some of the troubling potential problems that the community has raised are resolved, if not fully addressed’ (P10—Advocate, PLHIV Network). Rather than stopping MHS/CDR, another participant who worked in a jurisdiction that had conducted some MHS prior to the national mandate in 2018 wanted to slow the rollout to better document the impacts and work from there:

I’ve been in [high-level] meetings and everybody said ‘Hey, we don’t know the evidence’, but I would not say that’s a good argument to say, ‘Let’s not do it’, but I think let’s do it in some jurisdictions … and see what they find. I think we need the evidence to find that out, but it doesn’t mean you have to give a mandate to do it to everybody. It means you do it in some jurisdictions. (P24—Public Health Professional)

Debates and discussions about the quality of evidence supporting MHS/CDR programs show the health policy counterpublic at its most rational-critical and ‘scientistic’, with participants using the language of science, evidence, and the public interest to support positions advanced in the health policymaking arena (see Irwin, 2006; Welsh & Wynne, 2013; Wynne, 2006). This was also the arena in which the counterpublic was least unified, demonstrating the potential for heterogeneity within a health policy counterpublic.

### New matters of ethical concern: Challenging MHS/CDR in light of HIV criminalization, lacking consent affordances, communication norms, and digitization in public health

The emergence of the MHS/CDR health policy counterpublic involved opening up new opportunities for actors to reframe key issues in public health policy, ethics, and practice in relation to the perceived risks of MHS/CDR. Topics raised included HIV criminalization, consent in public health programs, the ethics of publishing or communicating information about MHS/CDR, and the digitization of health systems. The ways that the counterpublic addressed these issues offer examples of how it problematized critical areas of HIV public health policy in relation to routine MHS/CDR activities while also advocating for policy-level reforms.

In 2021, networks of people living with HIV published a report titled ‘Molecular HIV surveillance: A global review of human rights implications’, along with a video and press releases which argued that MHS/CDR posed the possibility of ‘a perfect storm’ in the context of HIV criminalization (HIV Justice Network, 2021; HIV Justice Network & Positive Women’s Network, 2020). The possibility of MHS amplifying issues related to criminalization was a significant matter of concern articulated within the health policy counterpublic. Many feared that data used for MHS/CDR could be accessed by law enforcement or in criminal prosecution of HIV non-disclosure or transmission (Hoppe et al., 2022; McClelland et al., 2020). As P07 (Advocate, PLHIV Network) put it: ‘Molecular HIV surveillance opens up one more potential tool in the toolbox to be used to prosecute and criminalize people living with HIV who are already extremely vulnerable and who already are living inside this transphobic, racist system.’ Discussion of criminalization often centered on whether the science of MHS could be used to determine directionality of transmission (e.g. person A transmitted HIV to person B), and whether this could be used in criminal proceedings. One participant explained:

The threat of criminalization is another reason why accepting MHS is hard. They will tell you, ‘We can’t prove directionality right now’, but I do know things are inferred and have been used in the legal context. We need to be cautious. (P17—Public Health Professional)

Participants differed in their beliefs about whether currently available phylogenetic methods could accurately determine or infer directionality of transmission. Some thought that the science would eventually advance to be able to definitively determine transmission directionality from ‘person A to person B’. It was pointed out that HIV criminalization laws center on either alleged ‘intent’ to transmit or ‘non-disclosure’ to partners, rather than actual HIV transmission. However, it was argued that phylogenetic evidence could still be interpreted to support criminalization, and cited cases where this had occurred, or broader examples in which contemporary law or policymaking did not reflect knowledge about how HIV is transmitted (e.g. laws that include spit as an HIV-transmitting fluid, which it is not). As P07 (Advocate, PLHIV Network) pointed out: ‘It doesn’t matter whether you can actually show who got HIV from whom, if a jury or if a judge is deciding on the basis of HIV hysteria.’ Participants advocated for reform of HIV criminalization laws: ‘I’m not saying that it would make all concern about MHS disappear, but I think it would make a huge dent’ (P20—Public Health Professional).

Beyond the direct impact of criminalization on individuals, there was also attention to how public communication about ‘emerging clusters’, including in published research, could contribute to other forms of stigmatization, possibly intersecting with criminalization concerns or other forms of harassment. ‘You publish this beautiful multimillion dollar clustering article with trans women in [city], and that further stigmatizes and singles out that community. Thinking about prevention and care and true engagement of those communities, that may be a setback’ (P08—Researcher). Similarly, another participant pointed to how media reporting could contribute to stigma in rural communities: ‘You put out a press release saying there is a cluster in a rural area of my state, and everybody is looking at everybody they think is queer and thinking it’s that person’ (P04—Public Health Professional). Others problematized how MHS/CDR could characterize people living with HIV as vectors of transmission, with assumed public benefits justifying dehumanizing frames. For example, P19 (Advocate, PLHIV Network) explained:

We are human beings trying to live wholly, but the fact that you can ignore our privacy rights, that folks can talk about us and do research around us without our consent or involvement, or without a thought of the impact that it has on our lives, is just incredible to me, but it follows with this idea of, ‘We’re public health threats and so you can do this for the public good.’

MHS/CDR represented a source of potential increased stigma, including through the way it contributed to framing people living with HIV as ‘vectors of disease’ or the potential for public communication or news stories about clusters in local communities to create stigma (Chung et al., 2019; Hastings, 2022; Shook et al., 2021). These concerns mobilized many of the advocacy efforts of the health policy counterpublic, both in terms of how MHS represented people living with HIV, and in highlighting injustices in the legal system.

Finally, while the health policy counterpublic formed directly to counter MHS/CDR, the controversy also opened up space to rethink some taken-for-granted norms related to consent, data security, and privacy that are made more visible in increasingly digitized public health where HIV data are managed and stored for numerous surveillance, prevention, and research re-uses (Bollinger et al., 2023; Buchbinder et al., 2022; Molldrem et al., 2023b; Smith et al., 2023; see also Ribes, 2017). Under current legal and ethics frameworks in the US and most other countries, public health agencies are generally permitted to collect and re-use data about HIV and other infectious diseases without patient consent (Molldrem & Smith, 2020). Many participants, particularly community advocates, argued that people living with HIV should be able to consent to their data being used for MHS/CDR, or at least afforded the opportunity to become informed about how their data are used: ‘I think you need my consent. You’re doing a whole bunch of stuff with my information that I did not consent to. Once assured of privacy concerns, I may support something like this’ (P19—Advocate, PLHIV Network). Similarly, P14 (Advocate, PLHIV Network) explained: ‘Ideally, [consent] would happen at the clinic encounter at the blood draw.’

It was also argued that being unknowingly and non-consensually entered into a CDR intervention as the result of having routine tests as part of receiving care introduced new social and ethical dynamics: ‘I was talking to a DIS [Disease Intervention Specialist] who said to me, “People are shocked. This is so personal and so stigmatized, and they had no idea that their information is going to the health department. You’d be pissed, too”’ (P05—Public Health Professional). While possibilities for consent were contested, participants collectively argued for greater community education and efforts to inform patients about how their data might be used, noting that neglecting to do so could heighten distrust in health systems (Smith et al., 2023). Demands for consent affordances, opt-outs, or plain-language notifications about MHS/CDR became a specific policy request of many actors in the MHS/CDR counterpublic, which were legitimized by PACHA (Molldrem et al., 2023a, Molldrem, Smith, & Subrahmanyam, 2023; PACHA, 2022).

In addition to consent, participants expressed concerns about growing data security and privacy issues in digitized health systems. P10 (Advocate, PLHIV Network) explained: ‘As technology has increased the capacity of the public health sector to get finer and finer data, it raises new issues of privacy, human rights, civil rights, and privacy protection. The ability to violate or override privacy has increased.’ Some community advocates were not confident that data would be adequately protected and pointed to potential risks of breaches or sharing data with law enforcement, immigration, or other institutions. Regarding data collected for MHS/CDR, P12 (Advocate, PLHIV Network) argued that ‘there is no guarantee that this information would be kept private, confidential, by the analyzing organization’, and P06 (Advocate, PLHIV Network) observed ‘I have only seen data breaches and increasing abuse of the data collected for these purposes.’

In contrast, some participants reported confidence about security, privacy, and that data would be used appropriately, even though they expressed other concerns about MHS/CDR: ‘I feel really clear about the fact that they deidentify everything before it is shared back with CDC, that our security measures are very intense, very good’ (P20—Public Health Professional). Others explained that while specific institutions or states had good laws and infrastructure for security and privacy, these differed greatly between jurisdictions and institutions (see also NASTAD, 2018). While all participants agreed that privacy and security were important, they held varied views regarding whether data was currently adequately protected. This shows the non-monolithic makeup of the MHS/CDR health policy counterpublic and the variety of perspectives held by the actors who constituted it. This open-ended structure is a crucial feature of counterpublics, because publics function as zones for ongoing debate and the exchange of views about key matters of concern as they develop during governance processes or specific controversies (Fraser, 1990; Irwin, 2006; Latour, 2004; Warner, 2002; Welsh & Wynne, 2013).

## Discussion: Ongoing controversies over HIV data

Understanding how controversies over health and science policy materialize and take shape, and how they effect change, requires attending to how social movements, (counter)publics, and different actors converge with, within, through, and against the state. We conceptualized health policy counterpublics as a specific type of constituency that can form against state-led health policies. This type of counterpublic is temporally bounded, holds an explicit desire to change policy in an area of interest, and succeeds in capturing the state’s attention. In this case study, a health policy counterpublic formed amongst networks of people living with HIV, researchers, ethicists, critical health scholars, state-employed public health personnel, and other advocates who drew on collective resources and infrastructures enabled by pre-existing social movements to challenge and reshape MHS/CDR policy. We focused on how this health policy counterpublic was enacted, and also the specific areas of controversy that emerged as matters of concern. These issues persist; since our interviews in 2021, there have been significant developments stemming from the actions of the MHS/CDR health policy counterpublic, including researchers pausing and then stopping a US National Institutes of Health-funded MHS study after consulting with communities (Tordoff et al., 2023), the previously-discussed October 2022 resolution from PACHA (PACHA, 2022), followed by a response directly from the White House Office of National AIDS Policy (ONAP) (Phillips, 2023). The MHS/CDR counterpublic has demonstrably affected the trajectory of US HIV/AIDS policy.

As with nearly all dominant publics and subaltern counterpublics (Fraser, 1990; Murphy, 2012; Warner, 2002), the MHS/CDR health policy counterpublic is far from homogenous. Many in our sample were not confident in the proposed benefits of MHS/CDR and mobilized arguments and scientistic forms of reasoning—the language of contemporary science policy (Irwin, 2006)—to suggest that the national mandated rollout should be halted or slowed until localized evidence could be generated, and risks addressed (Gore et al., 2021; Shook et al., 2021). Although some participants were hopeful that MHS/CDR could or did hold benefits for public health and ending the HIV epidemic, others challenged MHS/CDR as a distraction from what they perceived as more urgent priorities in addressing HIV. Amongst these participants, there was an argument that the disparities and gaps in the US HIV epidemic were relatively clear: They related to racial, economic, and health inequalities that could be understood through non-molecular epidemiological methods and addressed through structural investments in community prevention and health services (Bowleg et al., 2022; Philbin & Perez-Brumer, 2022). The pairing of structural critiques of the US healthcare system with specific arguments about perceived problematic MHS/CDR practices advanced by our participants and the broader MHS/CDR health policy counterpublic show how social movements can co-mobilize technical expertise and political analyses to achieve their goals, making appeals that fuse the social and political dimensions of a controversy to produce novel arguments about technoscience and its proper role in society (Irwin, 2006; Welsh & Wynne, 2013; Wynne, 2006).

In problematizing MHS/CDR, participants in our study raised substantive social, ethical, and policy challenges for the US and other jurisdictions that are potentially aiming to implement new forms of HIV public health surveillance (Garcia et al., 2023; Mutenherwa et al., 2019a; Schairer et al., 2019). These challenges included: a perceived erosion of vital relations of trust between HIV advocates and public health agencies; lack of clarity about benefits, impacts, and risks; increased stigma for already marginalized communities; increased risks of criminalization; and concerns about data rights and security. Aspects of these issues have also been explored through scholarly publics, mainly in the fields of bioethics, surveillance studies, and critical public health (Bollinger et al., 2023; Buchbinder et al., 2022; Molldrem et al., 2023b; Shook et al., 2021). Our analysis highlights that meaningfully engaging people living with HIV should be central in planning and implementing new and ongoing approaches in HIV prevention and surveillance (Bernard et al., 2020; HIV Justice Network & Positive Women’s Network, 2020; Tordoff et al., 2023). However, ‘meaningful engagement’ cannot be tokenistic, and is necessarily a question of who gets to enact programs, shape policy, and drive knowledge production (Smith et al., 2023; Spieldenner et al., 2022).

Notably, the MHS/CDR controversy arguably marks a new phase of the HIV social movement in relation to public health data practices. There has not been a controversy related to uses of HIV data in the US at the scale of the MHS/CDR conflict since debates about the introduction of names-based case reporting in the 1990s and 2000s (Fairchild et al., 2007). In the context of growing investments in big data and digital health, controversies over HIV data are likely to extend beyond this case: The matters of concern generated by the MHS/CDR health policy counterpublic were explicitly focused on broader issues in the political economy of health data (McClelland et al., 2020; Molldrem et al., 2023a, 2023b; Smith et al., 2023). Implementers of new policies related to HIV prevention and surveillance should consider how programs interact with existing HIV criminalization laws and practices, community trust in healthcare, stigma and discrimination, and data sharing, privacy, and security regulations.

## Conclusion: Emergent health policy counterpublics and health policy scenes

Health policy counterpublics offer a useful lens for health policy professionals and scholars engaged in sociotechnical studies of health policymaking to focus on how effective forms of dissent materialize from activities led by health-oriented social movements and their allies in the state, academia, civil society, and other sectors. Whether all health policy counterpublics exist in relation to pre-existing social movements is an open question that we cannot resolve. However, pre-existing social movements, spaces for deliberation and debate among members of those social movements, as well as the infrastructures provided by those movements and constituent organizations, were all crucial to the emergence of the MHS/CDR health policy counterpublic. The story of the MHS/CDR counterpublic’s development was thus as much about its specific policy aims and makeup as about the broader milieu from which it sprung—the politicized zone of HIV/AIDS policymaking, a scene in which many of the issues that the counterpublic would problematize were already partly pre-signified as the result of earlier controversies (Epstein, 1996, 2007; Fairchild et al., 2007). The MHS/CDR controversy was no one’s ‘first rodeo’, and many of the key players on all sides knew each other (or each other’s organizations) from earlier HIV policy dramas. The actors were part of a shared health policy scene that structures the ever-changing war of position in the governance of HIV.

We conclude with a note about the territories or scenes upon which conflicts over health policy unfold—the arrangements of power and institutions which give rise to conditions that enable, for example, forms of interwoven ‘insider’ and ‘outsider’ advocacy that the MHS/CDR health policy counterpublic has engaged in. These arrangements are distinctly contemporary. As Warner notes, ‘The idea of a public is a cultural form, a kind of practical fiction, present in the modern world in a way that is very different from any analogues in other or earlier societies’ (2002, p. 8). Publics and counterpublics require not only an audience but also means, platforms, and circuits of discursive circulation that enable the dissemination of their messages and their growth. To take shape, they require a shared cultural grammar and at least some common coordinates. The conditions of possibility that allowed the MHS/CDR health policy counterpublic to form have included conferences, strategy meetings, publications, social movement infrastructures, protests, news media, social media, and official policymaking processes in which individuals and groups have participated in both confrontational and rational-critical modalities. What was required was not only a committed group of people and organizations (although those were certainly necessary preconditions), but also an entire policy scene on which the actors in the counterpublic could stage collective acts of insider and outsider policymaking performances that explicitly sought to achieve specific goals.
